# Emerging Translational Research in Neurological and Psychiatric Diseases: From In Vitro to In Vivo Models

**DOI:** 10.3390/ijms242115739

**Published:** 2023-10-30

**Authors:** Masaru Tanaka, Ágnes Szabó, László Vécsei, Lydia Giménez-Llort

**Affiliations:** 1 Danube Neuroscience Research Laboratory, HUN-REN-SZTE Neuroscience Research Group, Hungarian Research Network, University of Szeged (HUN-REN-SZTE), Tisza Lajos krt. 113, H-6725 Szeged, Hungary; vecsei.laszlo@med.u-szeged.hu; 2 Department of Neurology, Albert Szent-Györgyi Medical School, University of Szeged, Semmelweis u. 6, H-6725 Szeged, Hungary; szabo.agnes.4@med.u-szeged.hu; 3 Doctoral School of Clinical Medicine, University of Szeged, Korányi fasor 6, H-6720 Szeged, Hungary; 4 Institut de Neurociències, Universitat Autònoma de Barcelona, Cerdanyola del Vallès, 08193 Barcelona, Spain; 5 Department of Psychiatry & Forensic Medicine, Faculty of Medicine, Campus Bellaterra, Universitat Autònoma de Barcelona, Cerdanyola del Vallès, 08193 Barcelona, Spain

## 1. Introduction

Revealing the underlying pathomechanisms of neurological and psychiatric disorders, searching for new biomarkers, and developing novel therapeutics all require translational research [1]. In vivo and in vitro models have been instrumental in elucidating complex polygenic, multifactorial, and heterogeneous mechanisms [2]. In recent years, advanced preclinical models have revealed the intriguing interaction of sex/gender and aging with the pathogenesis and clinical manifestations of neuropsychiatric disorders [3,4,5]. However, despite these advancements, a great deal of work remains to be undertaken to fully comprehend the underlying pathology of these conditions and to elaborate treatments that can significantly improve the lives of those who suffer from them. The current challenge in the field of neurological and psychiatric disorders is the development of disease-modifying, effective treatments for these complex and long-lasting debilitating conditions with a high burden of malady.

The first edition of this research topic, entitled ‘Emerging Translational Research in Neurological and Psychiatric Diseases: From In Vitro to In Vivo Models’, reinforces the notion that translational research plays a critical role in bridging the gap between basic research and clinical applications. In addition, it provides a platform for investigators to share their findings and advancements in translational research in this field. This new collection gathers 25 papers offering insights into the latest advancements in translational research and potential new avenues for treatments. These papers address a range of topics, including engineering novel preclinical models, utilizing in vitro and in vivo methods, and applying qualitative and quantitative research techniques.

## 2. Topic Articles

### 2.1. Neurological Disorders and Therapies

#### 2.1.1. Pathophysiology

Understanding the pathogenesis of Alzheimer’s disease (AD) and elaborating suitable preclinical models are vital for research [6,7]. Castillo-Mariqueo et al. devise a behavioral observation method to study gait and exploratory activity during AD progression and aging, adding motor symptoms to the classical cognitive perspective. Their work demonstrates pronounced functional impairment in 3× Tg-AD mice, underscoring the model’s applicative potential in AD research and therapeutic development [8]. This study contributes to our understanding of AD’s complexities and potential treatment avenues, aligning with broader research in this field. Santana-Santana et al. investigate how the marble burying test outcomes differ between male and female mice, shedding light on potential sex-dependent variations in behavior relevant to aging and AD. The article also examines how these behaviors alter over time, providing insights into the progression of age- and AD-related behavioral changes in mice [9]. Furthermore, Lam et al. identify age-related disturbances in retinoids and sex hormones, particularly in the context of AD and Parkinson’s disease (PD) [10]. The study suggests that these molecules may play a pivotal role in the pathogenesis and progression of these debilitating neurological conditions. These insights are valuable for advancing our knowledge of the mechanisms implicated in AD and PD, and potentially paving the way for innovative therapeutic approaches [11,12].

It is essential to burgeon novel therapeutic strategies for neurological and cardiovascular diseases, which are leading causes of morbidity and mortality worldwide. Additionally, non-invasive brain stimulation techniques have become an integral aspect of clinical research in mental illnesses [13,14]. Methods such as transcranial magnetic stimulation and transcranial direct current stimulation have been employed in preclinical models to investigate their potential therapeutic alternatives [15,16]. These techniques offer a unique opportunity to modulate neural activity in specific brain regions, mimicking the neuromodulatory effects observed in human studies [17,18,19]. Non-invasive brain stimulation in preclinical research allows scientists to explore the neural circuitry involved in neuropsychiatric conditions, providing valuable insights into the underlying pathology. It also facilitates the assessment of the safety and efficacy of these techniques before translating them into clinical applications. Mitrečić et al. discuss the potential of stem-cell-based therapies, tissue engineering, and regenerative medicine in augmenting effective treatments for these diseases, emphasizing the criticality of interdisciplinary collaboration and the need for an enhanced understanding of the underlying pathomechanisms of these conditions [20]. Revealing iron metabolism in AD is crucial due to its significant role in brain function and the development of AD-related pathologies. Peng et al. review the recent advances made regarding the relationship between iron and AD, highlighting the importance of iron in the brain for treating AD and discussing the potential of iron chelators as a therapeutic option for AD [21].

Three papers contribute to our knowledge regarding the pathophysiology of AD and reveal potential therapeutic targets for the disease. Swingler et al. investigate the role of microRNA-455 in AD-related memory deficits and anxiety, highlighting potential targets for therapeutic intervention [22]. Sheikh et al. discuss the aggregation of cystatin C and its effect on protease activities and the formation of amyloid beta fibrils, which are key pathogenesis in AD [23]. In an in vitro model of AD, Fernandes et al. investigate the structural and functional alterations in mitochondria-associated membranes and mitochondria, elucidating the stress response mechanisms activated by the disease [24]. These papers collectively provide valuable information regarding the underlying pathology of AD and its potential therapeutic targets, which could aid in elaborating novel treatments for the condition.

#### 2.1.2. Therapies

Other researchers investigate the therapeutic potential of mesenchymal stem cells (MSCs) for neurological disorders. Fu et al. demonstrate that xenografts of human umbilical MSCs promote recovery after chronic ischemic stroke in rats [25]. The article highlights the potential of MSCs in treating chronic stroke and provides insights into the therapeutic benefits of the xenotransplantation of MSCs. Kassab et al. discuss the role of systemic filtering organs, particularly the kidney, in aging and rejuvenation from a systems biology perspective. It provides an overview of the major systemic causes of aging and identifies that the filtration system represents a clear gap in aging studies to date. The paper concludes by exploring possible rejuvenation avenues that must be developed in the future in order to address the complex topic of healthy aging [26]. These papers provide valuable information regarding the therapeutic potential of MSCs in treating neurological diseases and regenerative medicine, which can aid in elaborating novel therapeutic strategies for these conditions.

The common pathogenesis of neurodegeneration, such as inflammation, amyloid pathology, and microglial dysfunction, are explored in three articles that focus on AD and PD, two prevalent neurodegenerative diseases [27]. The articles also propose novel treatments that target these mechanisms. One article by Hsu et al. evaluates the effect of peiminine on PD by regulating the PINK1/Parkin pathway [28]. Another article by Tsay et al. assesses the effect of EK100 and Antrodin C on AD by enhancing microglial and perivascular clearance pathways [29]. A third article by Kuo et al. examines the role of neuron–microglia contacts in controlling PGE2 tolerance and the effect of inhibiting TLR4-mediated de novo protein synthesis on neurodegeneration [30]. Bezerra et al. also investigate the possible role of SerpinA1 in modulating transthyretin proteolysis, a process involved in various neurodegenerative conditions, including AD and familial amyloid polyneuropathy [31]. These papers offer valuable insights into the potential application of new therapeutic strategies for neurodegenerative diseases, which can facilitate the development of effective treatments for these conditions.

Bellon et al. investigate the optimization of neurite tracing and the further characterization of neuronal-like cells derived from human monocytes [32]. Revealing the mechanisms underlying the differentiation of human circulating monocytes into neuronal-like cells is crucial for identifying novel therapeutic strategies for neurological conditions, as demonstrated by this study. The findings contribute to the growing body of research on the potential of circulating monocytes in human blood to be transdifferentiated into neuronal-like cells, which could lead to improved outcomes for patients with neurological disorders.

Transcranial alternating current stimulation (tACS) possesses the potential to reduce the symptoms of AD and improve cognitive function in those who have it. Jeong et al. examine the effects of tACS on long-term potentiation in transgenic mice with AD, which is an important process for learning and memory. The advantage of using tACS in this experiment is that its current can oscillate at a specific frequency and interact with the intrinsic oscillation of the brain [33]. The article highlights the applicative potential of tACS in treating AD-related cognitive impairments, which can aid in the development of novel therapeutic strategies for AD.

Chen et al. investigate the potential role of microRNA-124 in treating retinal vasoregression in neurodegenerative diseases [34]. The study highlights the significance of microRNA-124 in regulating microglial polarization, which is implicated in the retinal vasoregression. The findings contribute to our understanding of the potential of microRNA-124 in treating retinal vasoregression, aiding in searching novel therapeutic strategies for these conditions. The article also sheds light on the potential of microglial polarization as a therapeutic target for neurodegenerative diseases.

In a preclinical model of multiple sclerosis (MS), Quirant-Sánchez et al. investigate the application of a combined therapy approach involving vitamin D3-tolerogenic dendritic cells and interferon-β. The findings suggest that in a preclinical model, this combined treatment can effectively reduce the severity of MS symptoms and improve overall outcomes, potentially leading to the development of novel MS therapeutic strategies [35]. Thus, it is evident that the application of preclinical models has been instrumental in propelling research on MS forward [36]. These investigations have not only deepened our understanding of the intricate pathophysiology implicated in the condition, but have also been key in identifying potential biomarkers [37,38]. Furthermore, they have opened new avenues for the discovery of innovative treatments. Thus, the importance of these studies in shaping the future of MS research is undeniable.

### 2.2. Pain

Pain and mental illnesses are inextricably linked, and their comorbidities have been extensively investigated [39,40,41]. Neurogenic inflammation and neuropeptides have been implicated in the pathophysiology of various human diseases, including primary headache disorders and peripheral neuropathy [42]. These articles investigate the potential function of neurogenic inflammation and neuropeptides in the etiology and progression of a wide range of illnesses, from primary headache disorders to peripheral neuropathy [43]. The article by Spekker et al. discusses the impact of neurogenic inflammation on migraines and reviews recent findings from translational research on the subject [44]. A better understanding of its role in migraines could have crucial implications for the clinical management of this neurological condition. Employing the Class I HDAC inhibitor MS-275, Lamoine et al. provide vital new information regarding the benefits of using this drug to prevent chronic neuropathy and enhance antiproliferative activity in mice. The study utilizes a systems biology approach, combining transcriptomic and bioinformatic analyses to identify the molecular mechanisms underlying the effects of MS-275 [45]. The article highlights the potential of systems biology approaches in identifying novel therapeutic targets and elaborating more effective treatments for various diseases.

### 2.3. Psychiatric Disorders, Pathophysiology, Biomarkers, Therapies

Five articles highlight the significance of revealing the pathomechanisms underlying various conditions, including autism, sleep disturbance, and metabolic dysfunction, in searching novel therapeutic strategies. The role of the cerebellum and striatum in autism spectrum disorders is investigated by Thabault et al. This study is valuable because it investigates the neurological aspects of autism spectrum disorder, elucidates potential pathophysiology, and provides a link between clinical observations and preclinical models [46]. Lee et al. investigate the influence of maternal immune activation on male rat offspring. In this study, maternal immune activation is associated with social behavior deficits and hypomyelination, a condition characterized by reduced myelin in the brain, according to the study. These effects are observed in male rat offspring, and the investigation suggests that they have an autism-like microbiota profile [47]. Abuaish et al. investigate the potential of fecal transplant and Bifidobacterium treatments in modulating gut Clostridium bacteria and rescuing social impairment and hippocampal brain-derived neurotrophic factor expression in a rodent model of autism [48]. The article highlights the significance of understanding the role of gut microbiota dysbiosis in the pathophysiology of autism and the potential of microbiota-based interventions in scrutinizing for novel therapeutic strategies for autism.

Sleep is an integral component of energy metabolism, and sleep disturbance has been implicated in a wide range of metabolic disorders. Wei et al. provide a balanced overview of adipokines and their role in sleep physiology and sleep disorders with reference to recent human and preclinical studies [49]. The significance of this review lies in its contribution to our understanding of the relationship among sleep disturbance, metabolic dysfunction, and adipokines, which can aid in identifying novel therapeutic strategies for metabolic disorders. Garro-Martínez et al. investigate the potential role of mTOR expression in the infralimbic cortex in the pathophysiology of depression [50]. The article highlights the significance of revealing the mechanisms underlying mTOR expression in the infralimbic cortex and its potential role in the development of depressive-like behaviors. The findings contribute to the growing body of research on the potential of mTOR as a therapeutic target for depression, potentially leading to improved outcomes for individuals with this condition.

## 3. Conclusions and Future Directions

In vitro- and in vivo-based preclinical research serves as a vital complement to human studies in understanding neuropsychiatric conditions [51,52,53,54,55]. These models enable researchers to simulate disease conditions and explore the intricate connections among genetics, environment, pharmacology, and comorbidities [56,57,58,59,60]. This study provides insights into the pathomechanisms underlying neurological and mental disorders, facilitates the testing of potential treatments, and evaluates therapeutic efficacy [61]. For instance, studies in translational research illustrate how preclinical models aid in translating findings from the lab to clinical applications. Preclinical models have also been crucial in exploring neurological and psychiatric conditions like AD and autism spectrum disorder, and shedding light on their underlying factors. Furthermore, this approach contributes to the elaboration of personalized medicine by enabling the application of tailored treatments for mental disorders. It also enables the investigation of structural changes in the brain and the advancement of imaging techniques for clinical use. Preclinical research plays an essential role in unraveling the complexities of brain illnesses, offering valuable insights and testing treatments, and paving the way for innovative therapeutics and personalized medicine.

In this multidisciplinary endeavor, neuropharmacological research plays a pivotal role. The study of how drugs and compounds interact with the intricate neural networks present in preclinical models provides a deeper understanding of potential therapeutic agents [62,63,64,65,66,67,68,69]. These insights guide the future development of pharmacological interventions that can target the specific molecular pathways implicated in neuropsychiatric conditions. Researchers are exploring novel drug candidates, investigating their safety profiles, and assessing their efficacy in mitigating the symptoms of conditions like depression and anxiety, and the cognitive impairments associated with mental illnesses [70]. Advanced imaging techniques have significantly aided research on neuropsychiatric disorders. According to neuroimaging research, these conditions are associated with changes in brain structure and function [71,72,73,74,75,76,77,78]. These imaging methods can aid in the diagnosis of rare clinical cases and shed light on the underlying pathophysiology of the disorders being studied. Furthermore, neuropharmacological approach dovetails with the broader scope of preclinical investigations, facilitating a comprehensive exploration of the genetic, environmental, and pharmacological factors that influence mental health [66,68]. It expedites the identification of potential drug targets and the elaboration of personalized medicine approaches tailored to individuals’ unique neurochemical profiles [79,80,81].

In summary, we aspire for this subject to act as a pivotal platform for the exploration of the neural foundations of neurological and psychiatric disorders. Researchers are attempting to open novel avenues for specialized treatment plans and preventive measures, with the ultimate aim of improving the quality of life of those suffering from these complex mental health conditions, by examining behavioral neuroscience from this perspective [82,83,84,85,86,87,88]. As our comprehension of the pathomechanisms underlying neuropsychiatry advances, we draw nearer to a future in which individuals can receive personalized care and support to conquer these challenging conditions.

This comprehensive and interdisciplinary approach is echoed in various academic works and research endeavors, and serves as a valuable resource when hoping to comprehend the etiological factors of neuropsychiatric disorders, search for biomarkers, achieve precision, and master their personalized treatment. Additionally, discussions regarding the quest for neuropsychiatric biomarkers and endophenotypes are ongoing in academia [89]. Philosophical perspectives on neuropsychiatric topics are also being investigated, thereby contributing to our philosophical comprehension of psychology [90]. Research in this area often involves the examination of abstracts and articles, as exemplified by the National Institutes of Health’s database. This collective effort and interdisciplinary collaboration underscore the importance of advancing our understanding of mental illnesses and working towards enhanced treatments and support for affected individuals. We wish to express our heartfelt appreciation to all of those who contributed to this collection, and extend our gratitude to the reviewers for their invaluable feedback. We eagerly await future contributions that will further propel the fields of neurology and psychiatry, recognizing that your unwavering support and dedication play an indispensable role in shaping the progress and potential of this rapidly expanding domain.

## Data Availability

Data sharing is not applicable to this article.

## Abbreviations

AD	Alzheimer’s disease
MS	multiple sclerosis
MSCs	mesenchymal stem cells
PD	Parkinson’s disease
tACS	Transcranial alternating current stimulation
